# Waning humoral immune responses to inactivated SARS-CoV-2 vaccines in patients with severe liver disease

**DOI:** 10.1038/s41392-022-01032-9

**Published:** 2022-06-02

**Authors:** Zhiwei Chen, Yingzhi Zhang, Rui Song, Lu Wang, Xiaoxiao Hu, Hu Li, Dachuan Cai, Peng Hu, Xiaofeng Shi, Hong Ren

**Affiliations:** Key Laboratory of Molecular Biology for Infectious Diseases (Ministry of Education), Institute for Viral Hepatitis, Department of Infectious Diseases, the Second Affiliated Hospital of Chongqing Medical University, Chongqing, China

**Keywords:** Vaccines, Vaccines

**Dear Editor**,

The Coronavirus disease 2019 (COVID-19) pandemic caused by severe acute respiratory syndrome coronavirus 2 (SARS-CoV-2) is still ongoing around the world. Patients with severe liver disease (SLD), such as compensated cirrhosis (CC), decompensated cirrhosis (DC) or hepatocellular carcinoma (HCC), are highly vulnerable and have worse outcomes from COVID-19^1^. Vaccines are effective measure for the prevention of SARS-CoV-2 infection, severe disease, and mortality. Recent studies have preliminarily described the safety and immunogenicity of SARS-CoV-2 vaccines in patients with nonalcoholic fatty liver disease (NAFLD)^2^, and in patients with chronic hepatitis B virus infection (CHB)^3^. However, data are limited on the safety and immunogenicity of inactivated vaccines against SARS-CoV-2 in SLD patients. Additionally, the memory B cells (MBCs) responses and immunological memory after vaccination in SLD patients is still unclear. Hence, we aim to explore the safety, antibody responses, and MBCs responses of inactivated vaccines (BBIBP-CorV or CoronaVac) in SLD patients through a prospective observational study (NCT05007665).

A total of 334 participants were recruited from July 2021 to November 2021. Among them, 142 were healthy controls and 192 were SLD patients, including 82 CC, 45 DC, and 65 HCC. Most of the patients (88%, 169/192) were infected with HBV (Supplementary Table 1). And no participants occurred breakthrough infection during our observation period. Safety was assessed by determining the incidence of adverse events (AEs) via a standardized questionnaire. All subjects provided a blood sample at a single time point, between 21 and 105 days after full-course vaccination. Plasma samples were examined for receptor-binding domain IgG antibody (anti-RBD-IgG) and neutralizing antibodies (NAbs). MBCs responses were analyzed by flow cytometry. Detailed information is available in Supplementary Methods.

Firstly, we analyzed the safety of inactivated vaccines in SLD patients. The overall incidence of AEs within seven days in SLD patients was significantly higher than healthy controls (33.3% vs. 12.0%, *p* < 0.001) (Supplementary Table 2). The common AEs (>5%) in SLD patients in local AEs were pain at the injection site (28.7%) and itch (5.2%), in systemic AEs were muscle pain (6.8%) and fatigue (6.8%). The most common AEs in healthy controls was local pain at the injection site (6.3%). Subgroup analysis among CC, DC and HCC groups, the overall AEs incidence was the highest in HCC group, followed by DC group and CC group (46.2% vs. 31.1% vs. 26.8%). Most of AEs were mild and no vaccine related serious AEs was occurred, which was similar with previous studies^2,3^. Taken together, the SARS-CoV-2 inactivated vaccines in SLD patients were well tolerated.

Next, we evaluated the antibody responses in SLD patients. Although the overall seropositivity rate of anti-RBD-IgG was high in SLD patients (98.4% vs. 100%), geometric mean titers (GMTs) of anti-RBD-IgG in SLD patients were 1.4 times lower than in healthy controls (271.8 [95% CI 240.4–307.3 vs. 382.8 [333.5–439.4], *p* < 0.01) (Fig. 1a). Similarly, SLD patients had significantly lower detection level of NAbs (57.8% vs. 76.1%, *p* < 0.001), which was also lower than the seroprevalence that previous reported in NAFLD patients^2^ and CHB patients^3^. This indicates that SLD patients may have worse antibody responses than chronic liver diseases in general, such as NAFLD and CHB. Similarly, GMTs of NAbs was also reduced in SLD patients (4.84 [4.39–5.34] vs. 6.57 [5.69–7.59], *p* < 0.01). (Fig. 1a) Further analysis in CC, DC, and HCC groups, the results were in consent with the overall trend. Antibody titers in CC, DC and HCC groups were lower than healthy controls, especially in HCC group (anti-RBD-IgG: 215.5 [95% CI 175.3–264.9] vs. 382.8 [333.5–439.4], *p* < 0.001; NAbs: 4.45 [3.76–5.26] vs. 6.57 [5.69–7.59], *p* < 0.01). (Fig. 1b) Similar results were observed in gender, age, and vaccine types subgroup analysis (Supplementary Figs. 1–3). Additionally, the antibody responses in Child-Pugh classes B and C group were significantly lower than that in Child-Pugh class A group (Supplementary Fig. 4). In brief, the antibody responses to inactivated vaccines were inferior in SLD patients, especially in HCC group.

**Fig. 1:**
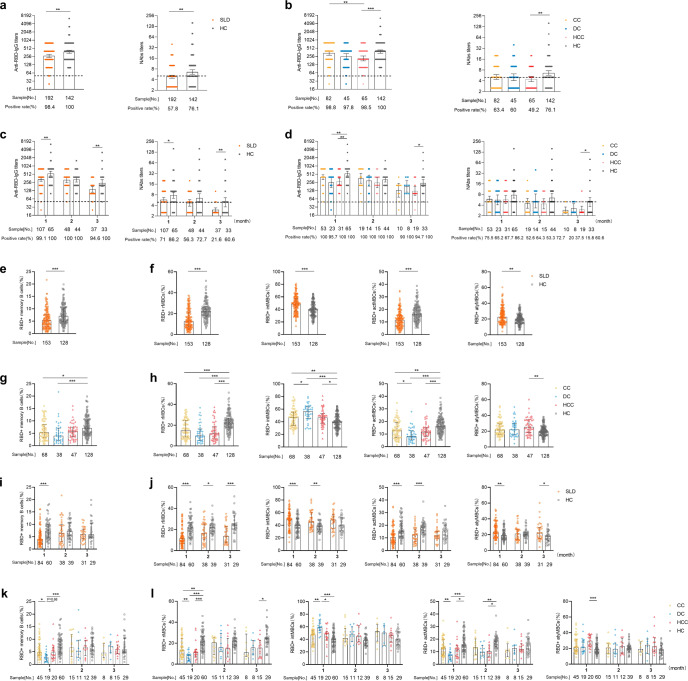
Humoral immune responses following immunization with inactivated vaccines in SLD patients. **a**–**d** Antibody responses after vaccinated with inactivated vaccines in SLD patients. The overall seropositivity rate and titers of anti-RBD-IgG (left panel) and NAbs (right panel) in SLD patients and healthy controls (**a**) and in CC, DC, HCC groups (**b**). The seropositivity rate and titers of anti-RBD-IgG (left panel) and NAbs (right panel) over time in SLD patients and healthy controls (**c**) and in CC, DC, HCC groups (**d**). The error bars in antibody titers indicate the 95% CI of the GMTs. Dotted lines indicate the detection limit. **e**–**h** RBD-specific MBCs responses after vaccinated with inactivated vaccines in SLD patients. The overall frequency (percentage of total B cells) of RBD-specific MBCs (**e**) and four subsets of RBD-specific MBCs (**f**) in SLD patients and healthy controls. The frequency of RBD-specific MBCs (**g**) and four subsets of RBD-specific MBCs (**h**) in CC, DC, HCC groups. **i**–**l** RBD-specific MBCs responses after vaccinated with inactivated vaccines in SLD patients with passing time. The error bars in **e**–**l** represent Median (IQR). ^*^p < 0.05, ^**^p < 0.01, ^***^p < 0.001. actMBCs, activated MBCs; anti-RBD-IgG, spike receptor-binding domain IgG antibody; atyMBCs, atypical MBCs; CC compensated cirrhosis; CI confidential interval; DC decompensated cirrhosis; GMTs geometric mean titers; HC healthy controls; HCC hepatocellular carcinoma; intMBCs, intermediate; IQR interquartile range; MBCs memory B cells; NAbs neutralizing antibodies; rMBCs resting MBCs; SLD severe liver diseases

To better understand the variation tendency of antibody responses with passing time, we stratified three groups (1-month, 2-month, and 3-month) by time interval after full-course vaccination (mentioned in Supplementary Methods). As shown in Fig. 1c, GMTs of anti-RBD-IgG in both groups were declined over time (327.2 [95% CI 280.0–382.4] vs. 539.2 [442.9–656.4] in 1-month [*p* < 0.01], 304.0 [240.6–384.1] vs. 325.9 [265.2–400.6] in 2-month and 137.5 [107.4–176.1] vs. 241.6 [180.8–322.9] in 3-month [*p* < 0.01]), which was similar with a previous study^4^. Notably, the seropositivity rate of NAbs was sharply reduced in SLD patients over time (86.2% vs. 71% in 1-month [*p* < 0.05], 72.7% vs. 56.3% in 2-month and 60.6% vs. 21.6% in 3-month [*p* < 0.01]). Further analyzed in CC, DC, and HCC groups, similar results were observed (Fig. 1d). The seropositivity rate of NAbs in HCC group were the lowest (67.7% in 1-month, 53.3% in 2-month, and 15.8% in 3-month). Altogether, the antibody responses in SLD patients were reduced quickly than healthy controls over time. Hence, more concern should be taken on this special population.

Since we found the antibody responses in SLD patients were attenuated, we next to evaluate whether RBD-specific MBCs responses were impaired. Overall, the frequency (percentage of total B cells) of RBD^+^ MBCs was significantly lower in SLD group than in healthy group (5.2% vs. 7.1%, *p* < 0.001), which hints that the immune memory to SARS-CoV-2 may be dysfunctional (Fig. 1e). Interestingly, the percentage of resting MBCs (rMBCs) and activated MBCs (actMBCs) were dramatically declined in SLD group (*p* < 0.001). (Fig. 1f) This indicated that the immune reactivation after inactivated SARS-CoV-2 vaccination maybe impaired in SLD patients^5^. Further analysis in CC, DC, and HCC groups, the overall frequencies of RBD^+^ MBCs among the three groups were reduced than healthy controls, which was significantly lower in CC and DC groups (*p* < 0.01) (Fig. 1g). Similarly, the declined percentage of rMBCs and actMBCs subsets in CC, DC, and HCC groups were also observed (Fig. 1h). In a word, the durable humoral immunity after vaccination in SLD patients were weakened.

Lastly, we investigated the tendency of MBCs responses with passing time. Interestingly, unlike the gradually declined trend of antibody titers, the frequency of RBD^+^ MBCs in SLD patients was waved over time (Fig. 1i), which was similar to a previous study^4^. In addition, all the four subsets of MBCs were unaltered over time (Fig. 1j). Further analysis in CC, DC, and HCC groups, similar results of MBCs responses with passing time were observed (Fig. 1k, l). These results indicated that the impaired MBCs responses to inactivated vaccines in SLD patients were relatively stable over time.

There are several limitations in this study. First, most etiology of SLD patients in this study were due to hepatitis B, and more hepatitis C or alcoholic liver disease patients need to be further observed. Second, prior SARS-CoV-2 infection can’t be excluded in this study. Third, the baseline clinical data before vaccination of SLD patients is hard to collected integrally, which hinders us to know the baseline status of these patients further.

In summary, our research shows that the inactivated SARS-CoV-2 vaccines are well tolerated, and there are no vaccine-related serious adverse events in patients with severe liver disease. However, the antibody responses and durable humoral immune responses were weakened. Therefore, this special population should be given priority to vaccinate SARS-CoV-2 vaccines and booster doses.

## Supplementary information

Supplementary materials

Ethical Approval Document

## Data Availability

The data included in this study are available upon request from the corresponding author.
