# Supplementary material for: Waning humoral immune responses to inactivated SARS-CoV-2 vaccines in patients with severe liver disease
Source: Signal Transduct Target Ther. 2022 Jun 2;7:174. doi: 10.1038/s41392-022-01032-9 (PMC9160847; doi:10.1038/s41392-022-01032-9)
Supplement: Supplementary file 1 — Supplementary materials [file 41392_2022_1032_MOESM1_ESM.docx]

Supplementary Materials for

**Waning humoral immune responses to inactivated SARS-CoV-2 vaccines in patients with severe liver disease**

Zhiwei Chen^#^, Yingzhi Zhang^#^, Rui Song^#^, Lu Wang, Xiaoxiao Hu, Hu Li, Dachuan Cai, Peng Hu, Xiaofeng Shi, Hong Ren^*^

Key Laboratory of Molecular Biology for Infectious Diseases (Ministry of Education), Institute for Viral Hepatitis, Department of Infectious Diseases, the Second Affiliated Hospital of Chongqing Medical University, Chongqing, China

^#^ These authors contribute equally to this work.

^*^ Correspondence: Hong Ren (renhong0531@vip.sina.com).

This file includes:

Materials and Methods

Supplementary Table 1 to 3

Supplementary Fig 1 to 5

**Supplementary methods**

**Study population and design**

In this prospective study, all adult participants (>18 years) with established SLD or healthy controls were recruited consecutively since 1 July 2021. All participants samples were collected 21 to 105 days after the second dose of inactivated vaccine (BBIBP-CorV/CoronaVac) at the Second Affiliated Hospital of Chongqing Medical University. SLD was defined as an ICD code corresponding to a diagnosis of cirrhosis, liver failure, HCC, liver transplantation, decompensated liver disease.^1^ ICD codes used to define outcomes are listed in Table S3. Decompensated liver disease was defined as coding for esophageal varices, ascites, hepatorenal syndrome or hepatic encephalopathy. The SLD was diagnosed by clinical, biochemical, radiologic, endoscopic or biopsy. Key inclusion criteria for healthy controls were without the history of cirrhosis, HCC or hepatic decompensation including ascites, hepatic encephalopathy, or variceal bleeding; HBsAg negative, or HCV-Ab negative. For all participants, the following conditions were excluded: a) the history of SARS-CoV-2 infection; b) coinfection with HIV; c) pregnancy and d) autoimmune disease.

For all participants recruited in this study, AEs within 7days and 30 days were recorded by questionnaire. Demographic characteristics and clinical data were obtained by questionnaire or electronic medical record. At each visit time, serum was used to test the antibody responses and PBMCs was used to examined the B cell responses. All AEs were recorded and graded according to the scale issued by National Medical Products Administration of China (version 2019). AEs related to vaccination were judged by investigators. For serious AEs, we plan to monitor for up to 1 year.

Firstly, we conducted a cross-sectional analysis. All subjects provided a blood sample at a single time point, between 21 and 105 days after full-course vaccination. To better investigate the change of antibody and B cell responses over time, we defined the gap of 21-45 days as “1-month”, 46-75 days as “2-month”, and 76-105 days as “3-month”. For the participants at 1-month, we plan to continue follow up to 1 year. During the follow up period, they will complete the vaccine booster according the National Vaccination Plan in China.

This study was approved by the Ethics Committee of the Second Affiliated Hospital of Chongqing Medical University and conformed with the ethical guidelines of the Declaration of Helsinki. Written informed consent was obtained from all participants. This ongoing prospective study has been registered at ClinicalTrials.gov (NCT05007665).

**Assay for spike protein receptor binding domain IgG antibody**

Serum samples were taken 21-105 days after the second dose vaccination. According to the manufacturer's protocol, the indirect ELISA method was used to detect spike protein receptor binding domain IgG antibody (anti-RBD-IgG) (Sino Biological, Beijing, China). Briefly, 0.5 ug/mL recombinant RBD protein was pre-coated on the plate wells (100 μL per well) by incubation at 4 ℃ overnight. After thoroughly discarding solutions in the plate, 300 μL of 6% BSA solution was added to each well and incubated for 1 hour at room temperature. After washing wells thoroughly, serially diluted samples or controls (100 μL) were added, mixed well, and incubated for 2 hours at room temperature. Following three times of washing plates, diluted horseradish peroxidase (HRP) conjugated goat anti-human IgG secondary antibody was added (100 μL per well), mixed well, and incubated for 1 hour at room temperature. After washing and adding substrate solution (TMB) and then stop solution, absorbance (OD value) was read at 450 nm. Serum samples were diluted with two-folded serial dilution starting from 1:50. In each plate, serially diluted positive antibody controls (anti-RBD antibody) and negative controls (serum from individuals without a history of SARS-CoV-2 infection and vaccination) were detected simultaneously. ELISA measurements were performed in duplicate. According to the manufacturer's instructions, a serum was considered seropositive for IgG binding antibodies when OD value ≥ 2.1 times the mean absorbance value of negative controls at 1:50 dilutions. The antibody titers were presented as the highest serum dilution showing a positive result. The kit reported that anti-S-RBD-IgG tests have 100% sensitivity and 98% specificity for the diagnosis of COVID-19.

**Assay for the neutralizing antibodies**

Serum samples were taken 21-105 days after the second dose vaccination. The neutralizing antibodies (NAbs) activity to block the interaction between RBD and ACE2 was assayed by competitive ELISA according to the manufacturer's protocol (Sino Biological, Beijing, China). Briefly, microplates were coated with 1 μg/mL human ACE2 recombinant protein (100 μL per well) by incubation at 4 ℃ overnight. After thoroughly discarding solutions in the plate, 300 μL of 6% BSA solution was added to each well and incubated for 1 hour at room temperature. After washing wells thoroughly, serially diluted samples (50 μL) and RBD protein linked to HRP (RBD-HRP) (50 μL) were added at the same time, mixed well, and incubated for 30 min at room temperature. After washing and adding substrate solution (TMB) and then stop solution, absorbance (OD value) was read at 450 nm. Serum samples were diluted with two-folded serial dilution starting from 1:5. In each plate, RBD-HRP only control, serially diluted positive controls (SARS-CoV-2 Inhibitors) and negative controls (serum from individuals without a history of SARS-CoV-2 infection and vaccination) were detected at the same time. ELISA measurements were performed in duplicate. Inhibition rate was calculated as 100 - [(OD value of sample/ OD value of RBD-HRP only control) × 100%]. According to the manufacturer's instructions, a positive result for the NAb was determined when the inhibition rate was ≥ 20%. The NAb titers was presented as the highest serum dilution showing a positive result. The kit reported that NAbs tests have 100% sensitivity and 100% specificity.

**SARS-CoV-2 specific memory B cells responses**

For SARS-CoV-2 specific MBCs responses, biotinylated SARS-CoV-2 Spike RBD protein (Sino Biological, 40592-V08H2-B) was mixed with Streptavidin BV421 (Biolegend, 405225) at 4:1 molar ratio for one hour at 4℃ to obtain the antigen probe. According to the manufacturer's instruction, peripheral blood mononuclear cells (PBMCs) were isolated from heparinized whole blood by Histopaque (Sigma-Aldrich, 10771) density gradient centrifugation. After washed by FACS buffer (PBS＋2% FBS), PBMCs were then stained for 30 minutes at 4℃ using antigen probe (1:33.3) and the following conjugated antibodies: anti-human CD3 (300430, Biolegend, 1:50), anti-human CD19 (302212, Biolegend, 1:50), anti-human CD21 (354918, Biolegend, 1:50), anti-human CD27 (356406, Biolegend, 1:50). After staining, cells were rewashed and resuspended in a 200ul FACS buffer. Samples were then evaluated by flow cytometry (Beckman Coulter, CytoFLEX) and analyzed using FlowJo (Treestar, 10.0.7r2).

**Statistical analysis**

Appropriate methods were used for statistical analysis based on the type of data. For categorical variables, the Chi-Square test and Fisher's exact test were used. For continuous variables, the Mann-Whitney U test was used to compare two groups, and the Kruskal-Wallis test was used to compare three or more groups. All results of multiple comparisons were corrected using Bonferroni's correction. Spearman's rank correlation was applied for correlation between antibodies. Geometric mean titers (GMTs) and their corresponding 95% confidential interval (CI) were calculated based on the standard normal distribution of the log-transformed antibody titers. Categorical variables were presented as numbers (%), and continuous variables were presented as median (IQR). A two-sided p-value < 0.05 was considered statistically significant. SPSS (IBM, 24.0.0) was used for statistical analysis. Graphpad Prism (GraphPad Software Inc, 9.2.0) was used for plotting.

**Reference**

1. Hagström H, Talbäck M, Andreasson A, et al. Repeated FIB-4 measurements can help identify individuals at risk of severe liver disease. J Hepatol 2020;73(5):1023-29.

**Supplementary Tables**

**Supplementary Table 1. The demographic characteristics of participants.**

| Variables | SLD patients (n=192) | Healthy controls  (n=142) | P value | CC patients (n=82) | DC patients (n=45) | HCC patients  (n=65) |
| --- | --- | --- | --- | --- | --- | --- |
| Age (years), median (IQR) | 53 (47-59) | 48 (33-60) | <0.01 | 52 (47-57) | 53 (48-59) | 55 (47-62) |
| 18-59 years (n, %) | 146 (76) | 101 (71) | 0.312 | 68 (83) | 35 (78) | 43 (66) |
| ≥60 years (n, %) | 46 (24) | 41 (29) |  | 14 (17) | 10 (22) | 22 (34) |
| Gender (male/female), (n/n) | 138 /54 | 85 /57 | <0.05 | 55 /27 | 32 /13 | 51 /14 |
| Age & Gender |  |  |  |  |  |  |
| 18-59 years & male (n, %) | 112 (58) | 63 (44) |  | 49 (60) | 26 (58) | 37 (57) |
| 18-59 years & female (n, %) | 34 (18) | 38 (27) | 0.073 | 19 (23) | 9 (20) | 6 (9) |
| ≥60 years & male (n, %) | 26 (28) | 22 (15) |  | 6 (7) | 6 (13) | 14 (22) |
| ≥60 years & female (n, %) | 20 (10) | 19 (13) |  | 8 (10) | 4 (9) | 8 (12) |
| Days after 2^nd^ dose vaccination, median (IQR) | 41 (30-65) | 51.5 (33.0-73.8) | <0.05 | 38 (30.0-56.8) | 41.0 (32.0-64.0) | 49.0 (31.0-82.0) |
| 1-month (21-45 days) (n, %) | 107 (56) | 65 (46) |  | 53 (65) | 23 (5111) | 31 (48) |
| 2-month (46-75 days) (n, %) | 48 (25) | 44 (31) | 0.197 | 19 (23) | 14 (31) | 15 (23) |
| 3-month (76-105 days) (n, %) | 37 (19) | 33 (23) |  | 10 (12) | 8 (18) | 19 (29) |
| BMI, median (IQR) | 23.3 (21.2-25.8) | 24.0 (21.9-26.0) | 0.327 | 24.5 (22.5-26.4) | 23.2 (20.9-25.0) | 22.0 (21.2-24.6) |
| <24kg/m^2^ (n, %) | 110 (57) | 72 (51) |  | 37 (45) | 27 (60) | 46 (71) |
| 24-28kg/m^2^ (n, %) | 67 (35) | 53 (37) | 0.323 | 37 (45) | 17 (38) | 13 (20) |
| >28kg/m^2^ (n, %) | 15 (8) | 17 (12) |  | 8 (10) | 1 (2) | 6 (9) |
| Vaccine type |  |  |  |  |  |  |
| BBIBP-CorV, n (%) | 55 (29) | 56 (39) |  | 18 (22) | 15 (33) | 22 (34) |
| CoronaVac, n (%) | 127 (66) | 78 (55) | 0.102 | 58 (71) | 27 (60) | 42 (65) |
| BBIBP-CorV + CoronaVac, n (%) | 10 (5) | 8 (6) |  | 6 (7) | 3 (7) | 1 (2) |
| Etiology of liver diseases |  |  |  |  |  |  |
| HBV/HCV (n) | 169/2 | / |  | 80/0 | 38/1 | 51/1 |
| NASH/ALD (n) | 1/3 | / | / | 1/0 | 0/2 | 0/1 |
| Others (n) | 17 | / |  | 1 | 4 | 12 |
| Clinical stages |  |  |  |  |  |  |
| With extrahepatic metastasis, n (%) | 17 (28) | / | / | / | / | 17 (28) |
| Without extrahepatic metastasis, n (%) | 48 (72) | / |  | / | / | 48 (72) |
| Treatment types |  |  |  |  |  |  |
| Treatment naïve, n (%) | 28 (43) | / |  | / | / | 28 (43) |
| Chemotherapy, n (%) | 2 (3) | / | / | / | / | 2 (3) |
| Immunotherapy, n (%) | 10 (15) | / |  | / | / | 10 (15) |
| Others^#^, n (%) | 25 (39) | / |  | / | / | 25 (39) |

^#^ Others therapies including surgery, targeted therapy and transcatheter arterial chemoembolization therapy. SLD, severe liver diseases; CC, compensated cirrhosis; DC, decompensated cirrhosis; HCC, hepatocellular carcinoma; IQR, interquartile range; BMI, body mass index; NASH, non-alcoholic steatohepatitis; ALD, alcoholic liver disease. p < 0.05 was considered statistically significant.

**Supplementary Table 2.** **Adverse events of COVID-19 vaccination in participants**

| AEs within 7 days | SLD patients  (n=192) | Healthy controls  (n=142) | P value | CC patients (n=82) | DC patients (n=45) | HCC patients  (n=65) | P value |
| --- | --- | --- | --- | --- | --- | --- | --- |
| Overall AEs | 64 (33.3%) | 17 (12.0%) | <0.001 | 22 (26.8%) | 14 (31.1%) | 30 (46.2%) | 0.043 |
| Local AEs |  |  |  |  |  |  |  |
| Pain | 55 (28.7%） | 9 (6.3%) | <0.001 | 19 (23.2%) | 12 (26.7%) | 24 (36.9%) | 0.177 |
| Swelling | 5 (2.6%) | 2 (1.4%) | 0.713 | 0 | 0 | 5 (7.7%) | 0.007 |
| Redness | 0 | 1 (0.7%) | 0.425 | 0 | 0 | 0 | - |
| Itch | 10 (5.2%) | 1 (0.7%) | 0.049 | 4 (4.9%) | 3 (6.7%) | 3 (4.6%) | 0.891 |
| Induration | 0 | 0 | - | 0 | 0 | 0 | - |
| Systemic AEs |  |  |  |  |  |  |  |
| Muscle pain | 13 (6.8%) | 1 (0.7%) | 0.006 | 4 (4.8%) | 1 (2.2%) | 8 (12.3%) | 0.078 |
| Pruritus | 1 (0.5%) | 0 | 1.000 | 0 | 0 | 1 (1.5%) | 0.375 |
| Rash | 1 (0.5%) | 0 | 1.000 | 0 | 0 | 1 (1.5%) | 0.375 |
| Fatigue | 13 (6.8%) | 1 (0.7%) | 0.006 | 3 (3.6%) | 0 | 10 (15.4%) | 0.002 |
| Drowsiness | 2 (1.0%) | 2 (1.4%) | 1.000 | 0 | 1 (2.2%) | 1 (1.5%) | 0.443 |
| Dizziness | 3 (1.6%) | 0 | 0.363 | 1 (1.2%) | 1 (2.2%) | 1 (1.5%) | 0.909 |
| Headache | 1 (0.5%) | 0 | 1.000 | 1 (1.2%) | 0 | 0 | 0.510 |
| Rhinorrhea | 0 | 0 | - | 0 | 0 | 0 | - |
| Laryngeal pain | 0 | 0 | - | 0 | 0 | 0 | - |
| Fever | 2 (1.0%) | 0 | 0.510 | 2 (2.4%) | 0 | 0 | 0.258 |
| Chill | 0 | 0 | - | 0 | 0 | 0 | - |
| Cough | 0 | 1 (0.7%) | 0.425 | 0 | 0 | 0 | - |
| Inappetence | 6 (3.1%) | 0 | 0.087 | 0 | 1 (2.2%) | 5 (7.7%) | 0.027 |
| Abdominal pain | 7 (3.6%) | 0 | 0.056 | 2 (2.4%) | 1 (2.2%) | 4 (6.2%) | 0.414 |
| Abdominal distension | 4 (2.1%) | 0 | 0.222 | 0 | 0 | 4 (6.2%) | 0.018 |
| Diarrhea | 4 (2.1%) | 0 | 0.222 | 2 (2.4%) | 1 (2.2%) | 1 (1.5%) | 0.928 |
| Hepatalgia | 2 (1.0%) | 0 | 0.510 | 0 | 0 | 2 (3.1%) | 0.139 |
| Nausea | 5 (2.6%) | 0 | 0.138 | 2 (2.4%) | 0 | 3 (4.6%) | 0.325 |
| Chest distress | 3 (1.6%) | 0 | 0.363 | 2 (2.4%) | 0 | 1 (1.5%) | 0.570 |
| Constipation | 0 | 0 | - | 0 | 0 | 0 | - |

SLD, severe liver diseases; CC, compensated cirrhosis; DC, decompensated cirrhosis; HCC, hepatocellular carcinoma; AEs, adverse events. p < 0.05 was considered statistically significant.

**Supplementary Table 3. ICD codes used to define severe liver diseases.**

| **Diagnosis** | **ICD-10 (1997-)** | **ICD-9 (1987-1996)** | **ICD-8 (1969-1986)** |
| --- | --- | --- | --- |
| **Severe liver disease** | | | |
| Liver failure, acute or subacute | K72.0 | 570 | 570 |
| Ascites | R18.9 | 789.5 | 785.3 |
| Esophageal varices, bleeding | I85.0, I98.3 | 456.0, 456.20 | 456.0 |
| Esophageal varices, non-bleeding | I85.9, I98.2 | 456.1, 456.21 | 456.0 |
| Hepatorenal syndrome | K76.7 | 572.4 |  |
| Liver failure, chronic | K72.1 | 572.8 | 573 |
| Liver cirrhosis | K74.6 | 571.5 | 571.9 |
| Liver encephalopathy |  | 572.2 | 573.02 |
| Liver failure not otherwise defined | K72.9 |  |  |
| Portal hypertension | K76.6 | 572.3 | 571.9 |
| Hepatocellular carcinoma | C22.0 | 155.0 | 155.01 |
| **Procedure codes** |  |  |  |
| Liver transplantation | JJC00, JJC10, JJC20, DJ005, DJ006, JJC30, JJC40 | 5200 | 5200 |
| Laparocentesis | TJA10 | 4041 | 4041 |


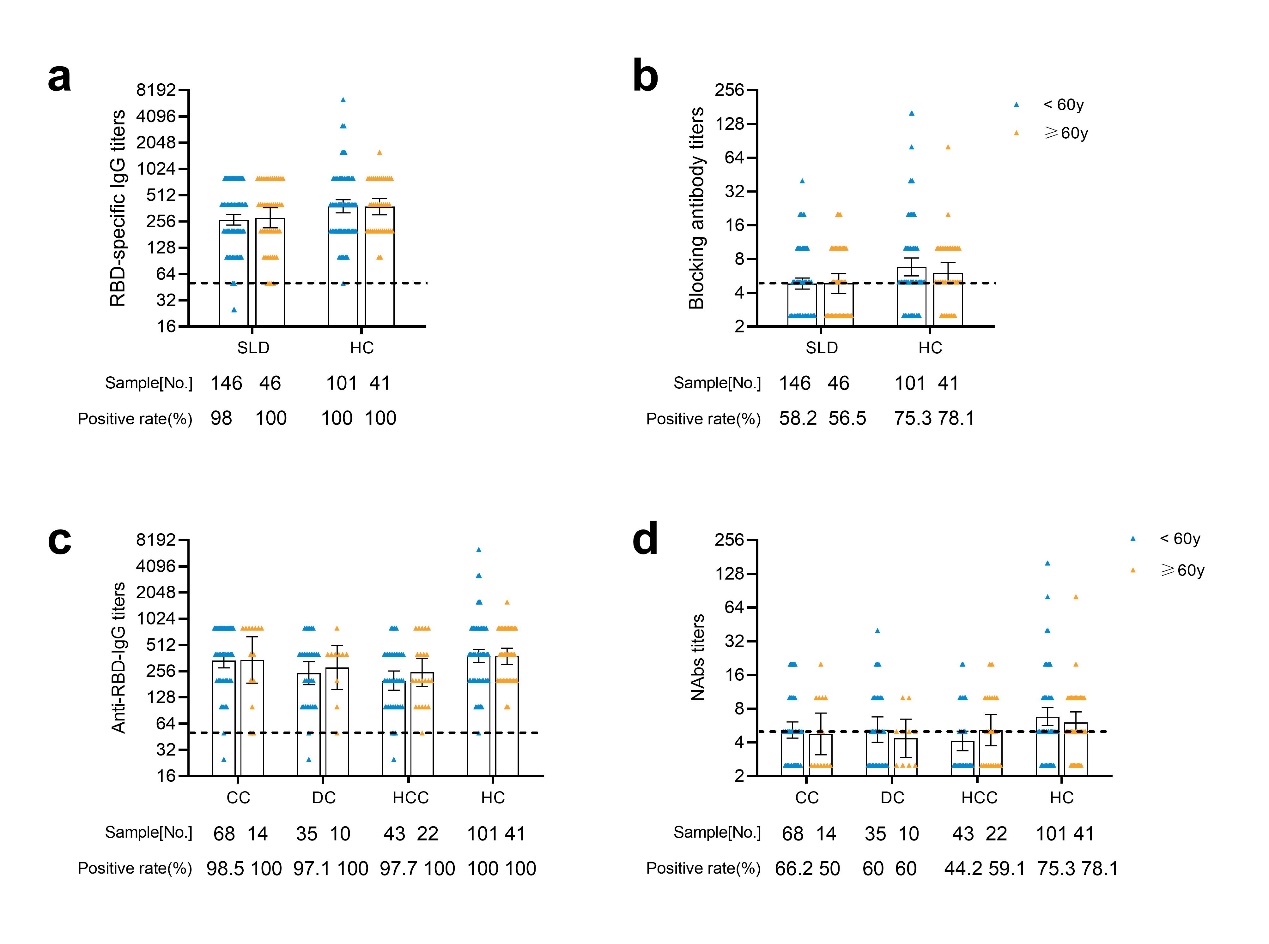


**Supplementary Figure 1.** Antibody responses to inactivated vaccines in older and young participants. **a-b** The seropositivity rate and titers of anti-RBD-IgG (**a**) and NAbs (**b**) in overall SLD patients and healthy controls by age. **c-d** The seropositivity and titers of anti-RBD-IgG (**c**) and NAbs (**d**) in CC, DC, HCC groups by age. Dotted lines indicate the detection limit. The error bars in antibody titers indicate the 95% CI of the GMTs. anti-RBD-IgG, spike receptor-binding domain IgG antibody; CC, compensated cirrhosis; CI, confidential interval; DC, decompensated cirrhosis; GMTs, geometric mean titers; HC, healthy controls; HCC, hepatocellular carcinoma; NAbs, neutralizing antibodies; SLD, severe liver diseases.


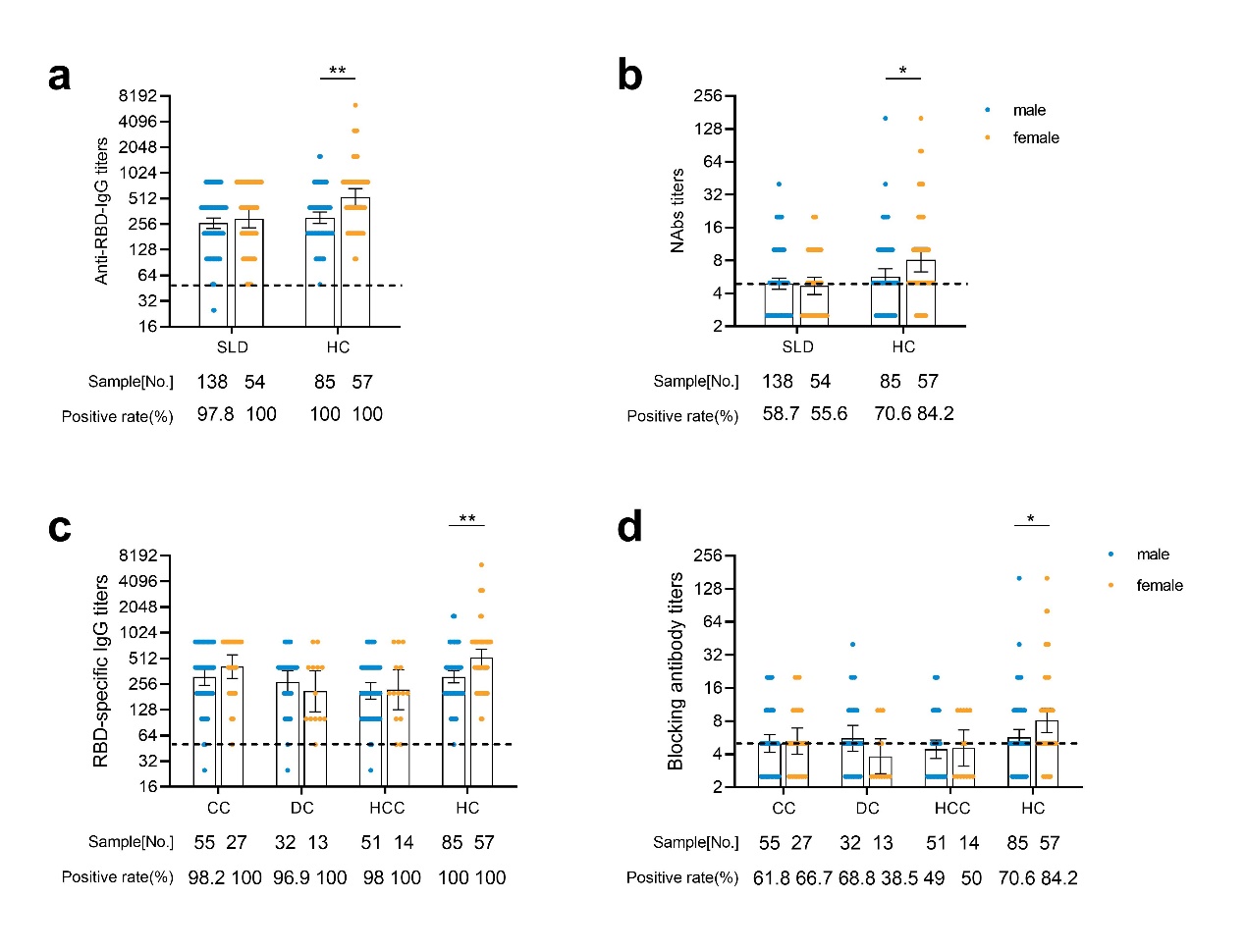


**Supplementary Figure 2.** Antibody responses to inactivated vaccines in male and female participants. **a-b** The seropositivity rate and titers of anti-RBD-IgG (**a**) and NAbs (**b**) in overall SLD patients and healthy controls by gender. **c-d** The seropositivity and titers of anti-RBD-IgG (**c**) and NAbs (**d**) in CC, DC, HCC groups by gender. Dotted lines indicate the detection limit. The error bars in antibody titers indicate the 95% CI of the GMTs. ^*^p<0.05, ^**^p<0.01. anti-RBD-IgG, spike receptor-binding domain IgG antibody; CC, compensated cirrhosis; CI, confidential interval; DC, decompensated cirrhosis; GMTs, geometric mean titers; HC, healthy controls; HCC, hepatocellular carcinoma; NAbs, neutralizing antibodies; SLD, severe liver diseases.


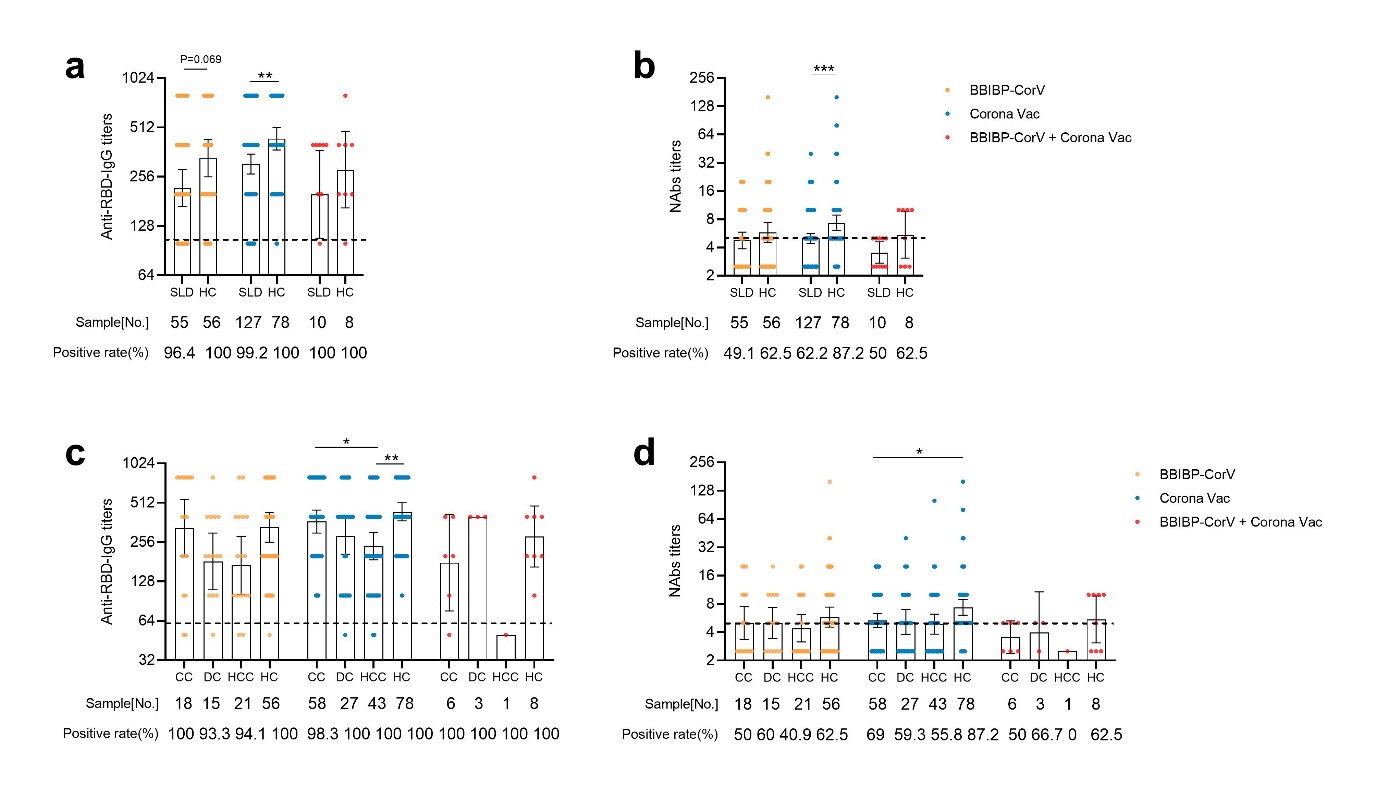
**Supplementary Figure 3.** Antibody responses to different types of inactivated vaccines in SLD patients and healthy controls. **a-b** The seropositivity rate and titers of anti-RBD-IgG (**a**) and NAbs (**b**) in BBIBP-CorV, CoronaVac and BBIBP-CorV + CoronaVac groups in overall SLD patients and healthy controls. **c-d** The seropositivity and titers of anti-RBD-IgG (**c**) and NAbs (**d**) in BBIBP-CorV, CoronaVac and BBIBP-CorV + CoronaVac groups in CC, DC, HCC groups by age. Dotted lines indicate the detection limit. The error bars in antibody titers indicate the 95% CI of the GMTs. ^*^p<0.05, ^**^p<0.01, ^***^p<0.001. anti-RBD-IgG, spike receptor-binding domain IgG antibody; CC, compensated cirrhosis; CI, confidential interval; DC, decompensated cirrhosis; GMTs, geometric mean titers; HC, healthy controls; HCC, hepatocellular carcinoma; NAbs, neutralizing antibodies; SLD, severe liver diseases.


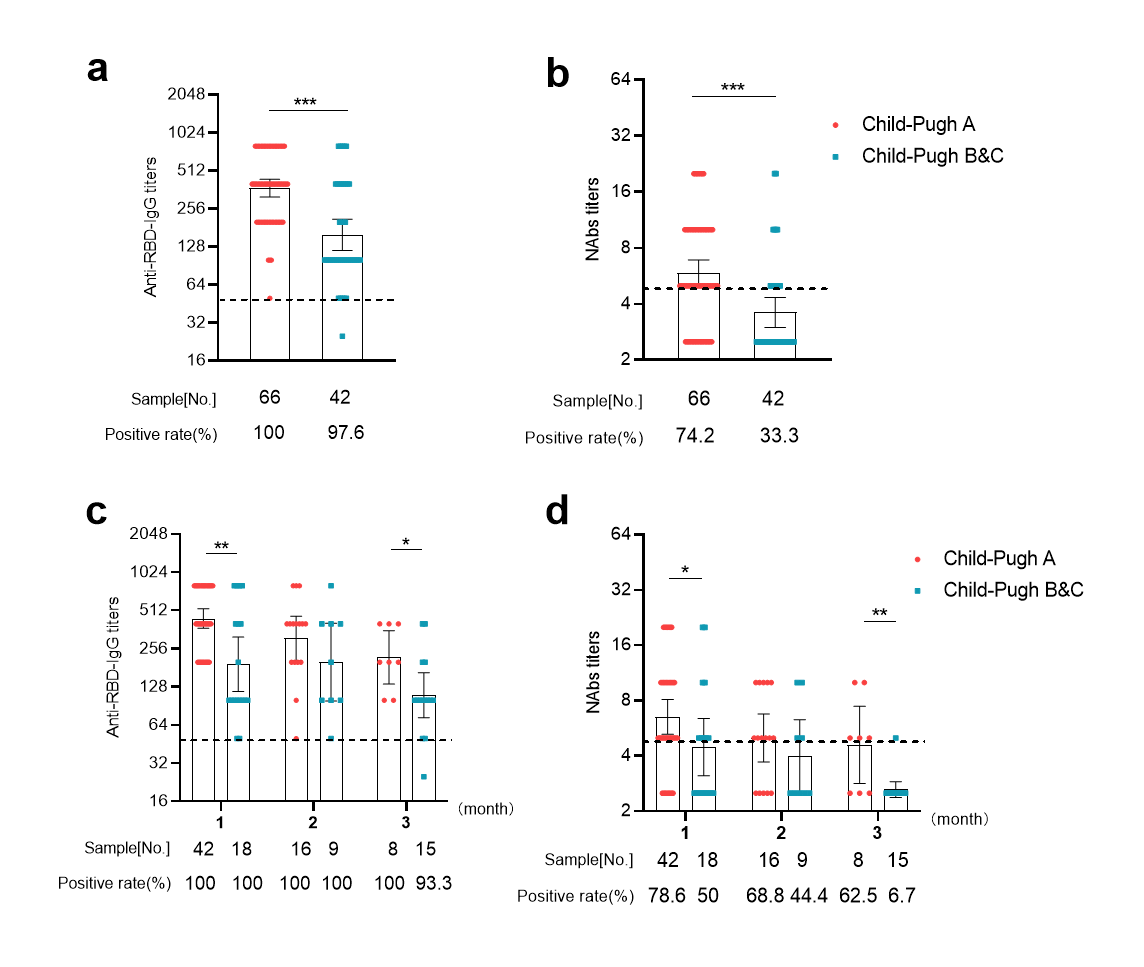


**Supplementary Figure 4.** Antibody responses to inactivated vaccines in SLD patients with Child-Pugh score. (**a-b**) The seropositivity rate and titers of anti-RBD-IgG (**a**) and NAbs (**b**) in SLD patients with Child-Pugh score. **(c-d)** The seropositivity and titers of anti-RBD-IgG (**c**) and NAbs (**d**) at 1-month, 2-month and 3-month in SLD patients with Child-Pugh score. The error bars in antibody titers indicate the 95% CI of the GMTs. ^*^p<0.05, ^**^p<0.01, ^***^p<0.001. anti-RBD-IgG, spike receptor-binding domain IgG antibody; CI, confidential interval; GMTs, geometric mean titers; NAbs, neutralizing antibodies; SLD, severe liver diseases.


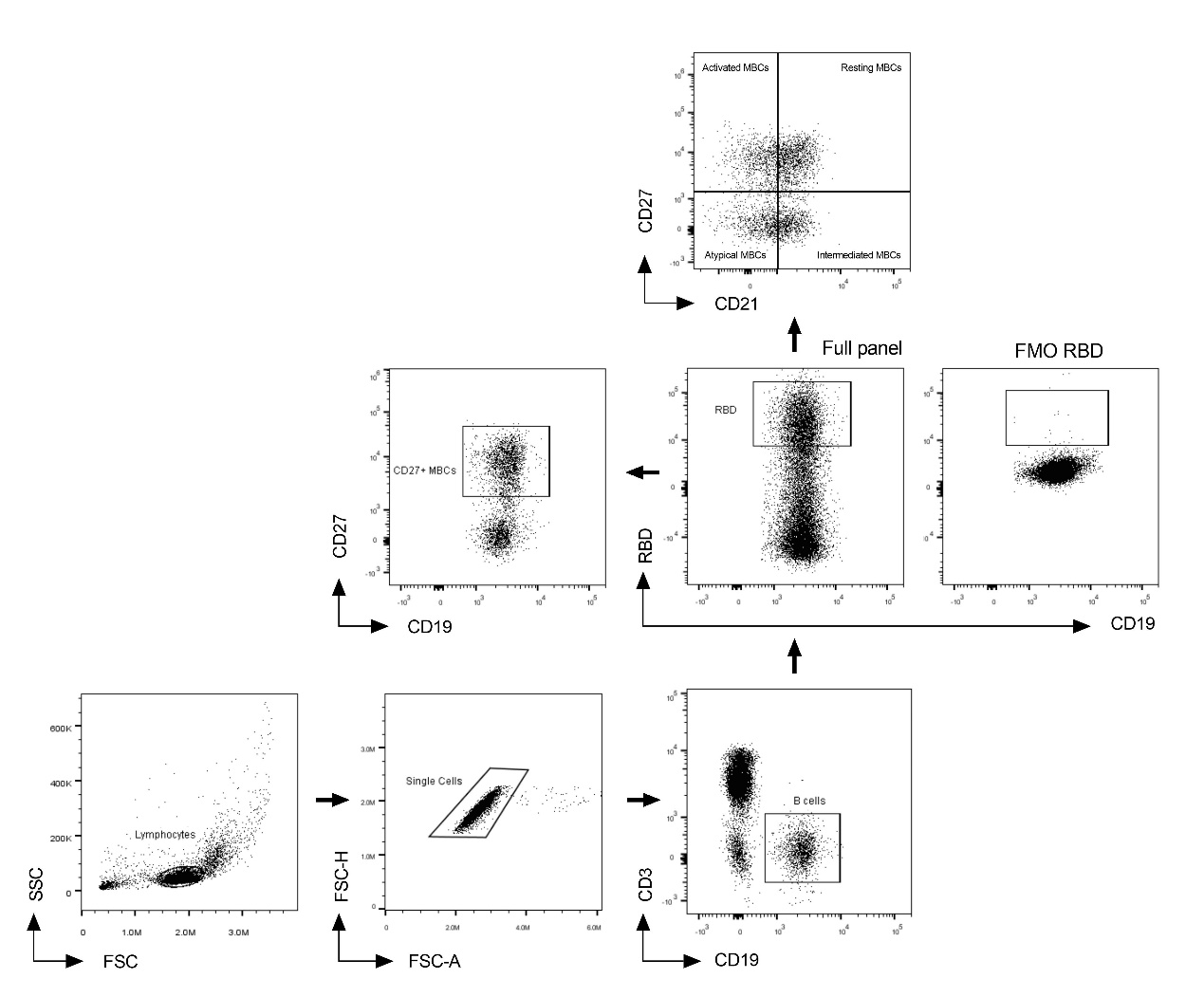


**Supplementary Figure 5.** Full gating strategy of flow cytometry for target cell population. RBD, receptor-binding domain; FMO, fluorescence minus one.
