# Supplementary material for: Waning humoral immune responses to inactivated SARS-CoV-2 vaccines in patients with severe liver disease
Source: Signal Transduct Target Ther. 2022 Jun 2;7:174. doi: 10.1038/s41392-022-01032-9 (PMC9160847; doi:10.1038/s41392-022-01032-9)
Supplement: Supplementary file 2 — Ethical Approval Document [file 41392_2022_1032_MOESM2_ESM.pdf]

重庆医科大学附属第二医院伦理委员会

重庆医科大学附属第二医院伦理委员会科研项目审查批件

2021 年科伦审第（ 94 ）号

审查项目名称	慢性肝病患者接种新冠疫苗安全性和有效性的探究		
科研课题来源	自拟课题		
申请科室	感染科	主要研究者	任红
审查方式	快速审查		
审核文件	1.伦理审查申请表<br>3.导师简历	2.研究方案<br>4.知情同意申请书	5.伦理递交函
伦理委员	性别	专业及职称	单位
王大刚	男	管理　高级经济师	重庆医科大学附属第二医院
任　红	男	内科学　教授	重庆医科大学附属第二医院
邓忠良	男	外科学　教授	重庆医科大学附属第二医院
胡怀东	男	内科学　主任医师	重庆医科大学附属第二医院
黄慧哲	男	生物化学　教授	重庆医科大学附属第二医院
殷跃辉	男	内科学　教授	重庆医科大学附属第二医院
杨刚毅	男	内科学　教授	重庆医科大学附属第二医院
熊正爱	女	妇产科学　教授	重庆医科大学附属第二医院
赵春景	男	药学　主任药师	重庆医科大学附属第二医院
王静	女	社区代表主任科员	重庆市渝中区解放碑街道办事处
林正伟	男	法学　高级律师	重庆市星全律师事务所
刘川	男	外科学　副教授	重庆医科大学附属第二医院
吴明珍	女	管理　副主任科员	重庆医科大学附属第二医院

意见	同意	作必要的修正后同意	不同意	终止或暂停已批准的试验
	√			

审评意见：1）任红委员回避此项目

2）经伦理委员会审核，该项研究申请者所提交的材料齐全。参加该项目审查的伦理委员根据有关法律/法规、赫尔辛基宣言等伦理原则对该项目提交的文件进行了审核，认为符合相关法律/法规，经表决，同意该课题的科研设计（包括受试者知情同意书）的内容，同意在我院开展本项研究。

医院伦理委员会（盖章）：重庆医科大学附属第二医院伦理委员会

日期：2021. 10. 20.

地址：重庆市渝中区临江门 76 号　邮编：400010　电话：02363693378
